# The Molecular Mechanism of Perillaldehyde Inducing Cell Death in *Aspergillus flavus* by Inhibiting Energy Metabolism Revealed by Transcriptome Sequencing

**DOI:** 10.3390/ijms21041518

**Published:** 2020-02-23

**Authors:** Chao Pan, Yong-Xin Li, Kunlong Yang, Erhunmwunsee Famous, Yan Ma, Xiaona He, Qingru Geng, Man Liu, Jun Tian

**Affiliations:** 1 College of Life Science, Jiangsu Normal University, Xuzhou 221116, China; tchaopan@outlook.com (C.P.); lyxycg@hotmail.com (Y.-X.L.); ykl_long@yeah.net (K.Y.); erhunfamous@yahoo.com (E.F.);; 2 Beijing Advanced Innovation Center for Food Nutrition and Human Health, Beijing Technology and Business University, Beijing100048, China

**Keywords:** *Aspergillus flavus*, *Perilla*, oxidative stress, energy metabolism, antifungal mechanism

## Abstract

Perillaldehyde (PAE), an essential oil in Perilla plants, serves as a safe flavor ingredient in foods, and shows an effectively antifungal activity. Reactive oxygen species (ROS) accumulation in *Aspergillus flavus* plays a critical role in initiating a metacaspase-dependent apoptosis. However, the reason for ROS accumulation in *A. flavus* is not yet clear. Using transcriptome sequencing of *A. flavus* treated with different concentrations of PAE, our data showed that the ROS accumulation might have been as a result of an inhibition of energy metabolism with less production of reducing power. By means of GO and KEGG enrichment analysis, we screened four key pathways, which were divided into two distinct groups: a downregulated group that was made up of the glycolysis and pentose phosphate pathway, and an upregulated group that consisted of MAPK signaling pathway and GSH metabolism pathway. The inhibition of dehydrogenase gene expression in two glycometabolism pathways might play a crucial role in antifungal mechanism of PAE. Also, in our present study, we systematically showed a gene interaction network of how genes of four subsets are effected by PAE stress on glycometabolism, oxidant damage repair, and cell cycle control. This research may contribute to explaining an intrinsic antifungal mechanism of PAE against *A. flavus*.

## 1. Introduction

*Aspergillus flavus* is a deadly saprophytic fungus distributed worldwide, and contaminates many important agricultural crops, such as maize, peanuts, and other crops [1]. Actually, *A. flavus* is the major producer of aflatoxin, a kind of highly carcinogenic toxins, which poses a dangerous health hazard to human beings and other animals [2]. Unfortunately, in 2004, a severe aflatoxicosis outbreak occurred in Kenya, resulting in 125 deaths because they unintentionally ate aflatoxin-contaminated maize [3]. Besides, *A. flavus* is also an opportunistic pathogen causing aspergillosis which is very fatal to immunocompromised patients [4]. Therefore, controlling contamination of *A. flavus* is a critical approach to ensure food safety and human health.

Perillaldehyde (PAE), a natural monocyclic terpenoid in Perilla plants, has a long history of usage as a flavoring ingredient in foods, and these foods seem to be completely safe with little or no health implications [5]. Interestingly, PAE shows an effectively antifungal activity. A volatility test using an air washer demonstrated that PAE displays the highest antifungal activity among five kinds of researched essential oils (EOs), of which three EOs belonged to aldehydes and two EOs contained a phenolic hydroxyl group [6]. In 2002, Mcgeady and coworkers found out that PAE had a biological activity of inhibiting the transformation of *Candida albicans* from a cellular yeast to filamentous and pathogenic cell type [7]. In 2015, we demonstrated that PAE may be used as a potential preservative agent in foods by two decay-inhibiting assays of cherry tomatoes and grapes against *A. flavus* and *Aspergillus niger*, respectively [8,9]. Besides the morphological evaluation of antifungal activity, an intrinsic antifungal mechanism was illuminated concentrating on a metacaspase-dependent apoptosis triggered by reactive oxygen species (ROS) accumulation induced by PAE [10]. In short, the primary mechanism of antifungal action of PAE has been investigated in previous study.

ROS accumulation triggering oxidant damage seems to be the core of antifungal mechanism of PAE against fungi. Furthermore, the apoptosis process is initiated by ROS through cytochrome c (Cyt c) catalyzing oxidation of cardiolipin (a polyunsaturated phospholipid in mitochondrial inner membrane), which leads to Cyt c release and apoptosis [11]. To combat oxidative stress, organisms have developed some antioxidant molecules, such as glutathione (GSH) and thioredoxin (Trx) [12]. GSH, the most abundant non-protein thiol, plays an important role in cellular defense against oxidant aggression, providing a protection against ROS damage-induced apoptosis [13]. Both GSH and Trx may be able to protect essential cellular components against oxidative modifications by their conserved cysteine residues drawing electrons from NADPH, eventually [14,15]. As we know, the pentose phosphate pathway (PPP) is a major source of nicotinamide adenine dinucleotide phosphate (NADPH). Besides, under a catalytic action of NADH kinase, NADPH also can be generated by a conversion from NADH, which is mainly produced during glycolysis [16]. Therefore, based on previous literature reviews, researched works and our previous study, we hypothesized that lower NADPH content produced by glycometabolism acts as the ultimate effector leading to *A. flavus* cell death induced by PAE. To shed more light on the energy-based relationship between antifungal action and *A. flavus* cell death, transcriptome sequencing of *A. flavus* treated with different concentrations of PAE was carried out. Understanding this antifungal mechanism will greatly help to improve control strategies in food preservation process.

## 2. Results

### 2.1. A Preliminary Analysis of Transcriptome Sequencing Data

To reveal an antifungal molecular mechanism of PAE against *A. flavus*, RNA-seq analysis was carried out. The spores of *A. flavus* were exposed to 0.5 µL/mL PAE, and samples were collected at 2, 4, and 6 h for transcriptome sequencing. All experiments were performed in triplicate, and samples not treated with oil were used as a control treatment; 45.98 Gb of clean data of 12 cDNA libraries were obtained, whereas over 3.15 Gb clean data of each library were gained. Moreover, clean base Q30 and mapped percent of each library were greater than 85% and 84%, respectively. All preliminary analysis data were shown in Table 1. Therefore, the sequencing data were of high quality, and suitable for further biological analysis.

### 2.2. Expression Patterns of DEGs

According to conventional threshold values of an adjusted *p*-value < 0.01 and absolute value of log_2_ FoldChange ≥1, differentially expressed genes (DEGs) between PAE groups and control treatment groups were preliminary screened. To eliminate lower expressed DEGs, the numerous mapped genes were filtered with a strict standard of FPKM > 1 at each time point after *A. flavus* spores were treated with PAE. Consequently, there were 2798, 2952, and 2954 DEGs filtered at corresponding time point. As for gene expression trends, approximately two-thirds of DEGs were upregulated, and one-third of DEGs was downregulated. Detailed analysis data were shown in Table 2.

**Figure 1:**
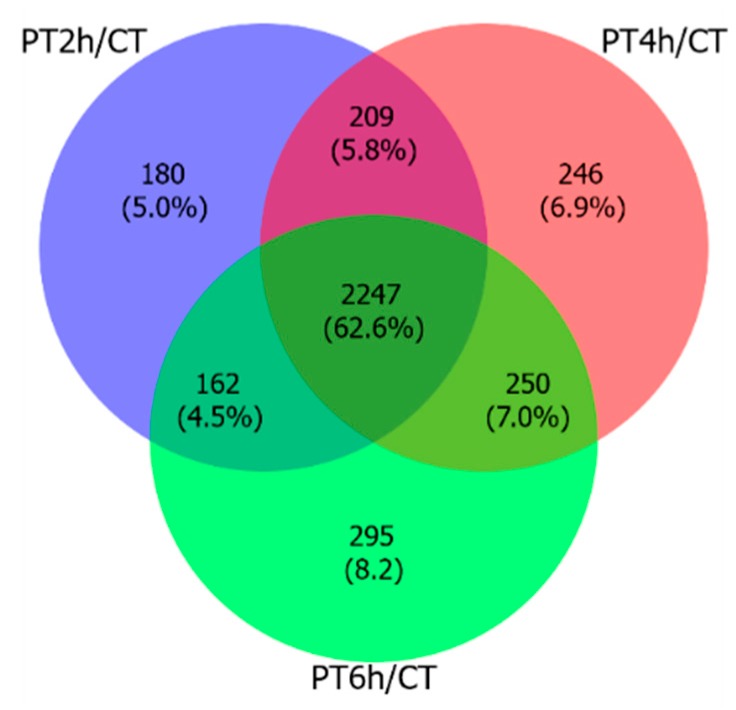
Venn diagram of differentially expressed genes (DEGs). This figure shows the number of common DEGs expressed in each PAE treatment group.

To screen critical gene sets in response to PAE antifungal stress, a Venn diagram was used to find the common DEGs (cDEGs) in all experimental treatment groups for subsequent analysis. There were 2247 cDEGs, and the number of specific DEGs in each PAE treatment group was increasing with extending treatment duration (shown in Figure 1).

**Figure 2:**
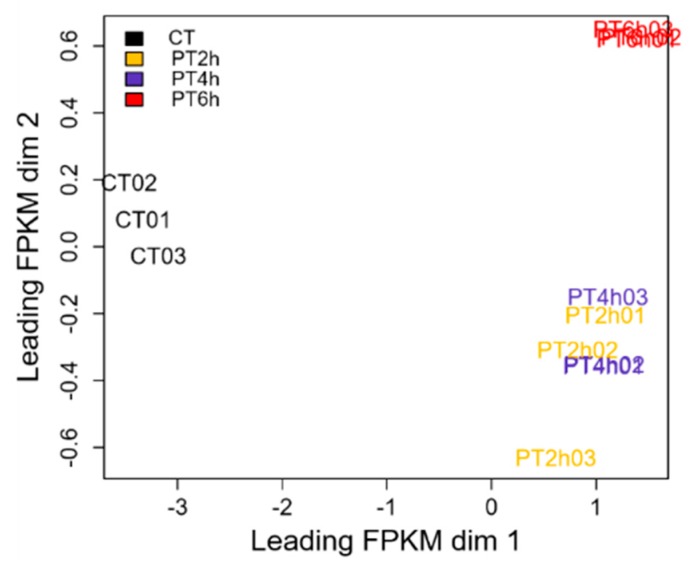
Multidimensional scaling plot of common differentially expressed genes (cDEGs). This figure shows expression pattern of gene set and data quality of cDEGs expressed in each experimental group.

The multidimensional scaling (MDS) plot looks similar to the principal component analysis, and can be used to check the gene expression profile and to evaluate data quality. MDS plot of cDEGs was drawn using FPKM at each experimental time point. As shown in Figure 2, the sequencing data of 4 and 6 h PAE groups and control groups exhibited remarkably high stability. However, data of 2 h PAE groups had a little dispersion, perhaps because PAE treatment time had an impact on a stable and adequate response of spores to PAE treatment. In general, this sequencing data reviewed a good group variation, and were suitable for further biological analysis.

**Figure 3:**
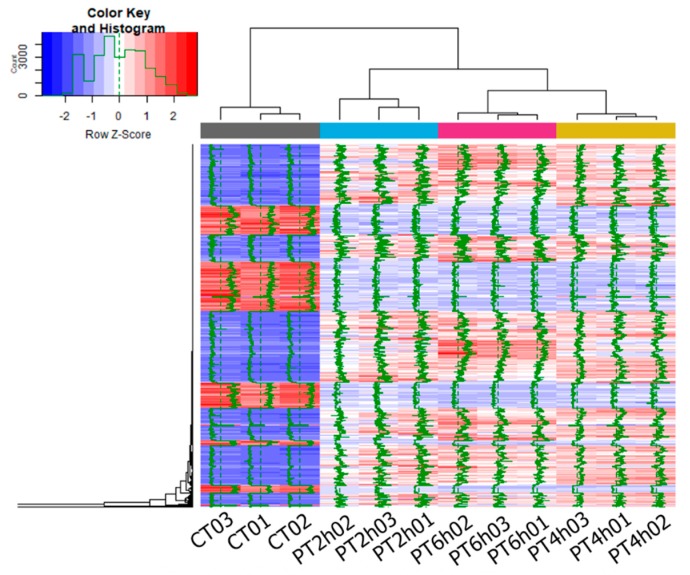
Heat map of DEGs. This figure shows gene expression profiles of common DEGs expressed in each experimental group.

A heat map of FPKM values (Figure 3) showed a marked variation between PAE treatment groups and control groups. Overall, FPKM values of low expressing genes in the control group increased remarkably after PAE treatment at different times, and in contrast, FPKM values of high expressing genes in the control group decreased sharply. This result revealed PAE has a significant effect on transcriptional level of response genes to PAE stress. Specific to PAE treatment groups of *A. flavus*, variation in the degree of gene expression data become larger and larger corresponding to increased PAE treatment time, according to mapping curve of FPKM values. However, an overall heat map is hard to identify key effective genes inducing *A. flavus* cell death. Therefore, enrichment analysis was carried out in further analyses to find out pivotal pathways associated with antifungal activity of PAE against *A. flavus*.

### 2.3. GO Enrichment Analysis of DEGs

**Figure 4:**
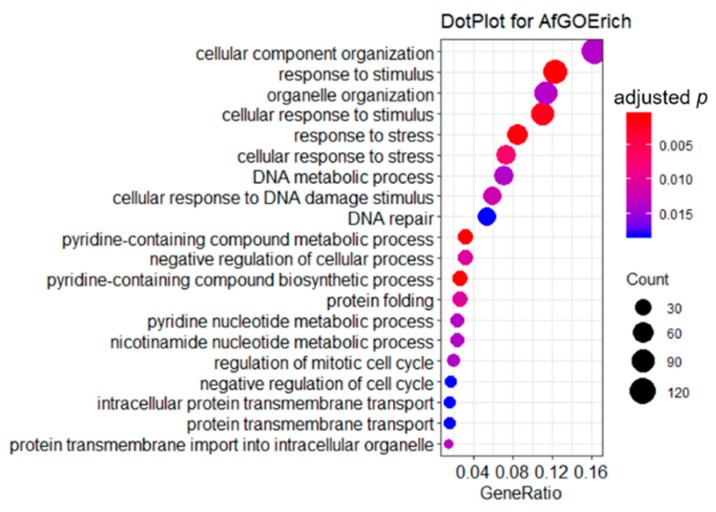
Enriched GO terms of DEGs. This figure shows the top 20 terms of GO enrichment analysis of common DEGs in PAE treatment and control groups.

GO enrichment analysis is a powerful method in analyzing gene expression profiles. Thousands of DEGs were enriched in 52 significant terms (adjusted *p*-values < 0.05), of which 49 were biological process terms and three were cellular component terms. The enriched terms were ranked by gene count, and then the top 20 terms were shown on a dot plot in Figure 4. The topmost significantly enriched GO terms in experimental groups were involved in PAE stress response, such as response to stimulus, cellular response to stimulus, response to stress, and cellular response to stress. In addition, some terms associated to DNA damage response also drew our great attention, such as DNA metabolic process, cellular response to DNA damage stimulus, DNA repair, and so on. In accordance with our previous study results, PAE was able to bring about ROS stress and DNA damage [10], resulting in cell death of *A. flavus*. Therefore, the response to stress term, a representative term was given detailed analysis in subsequent text.

**Figure 5:**
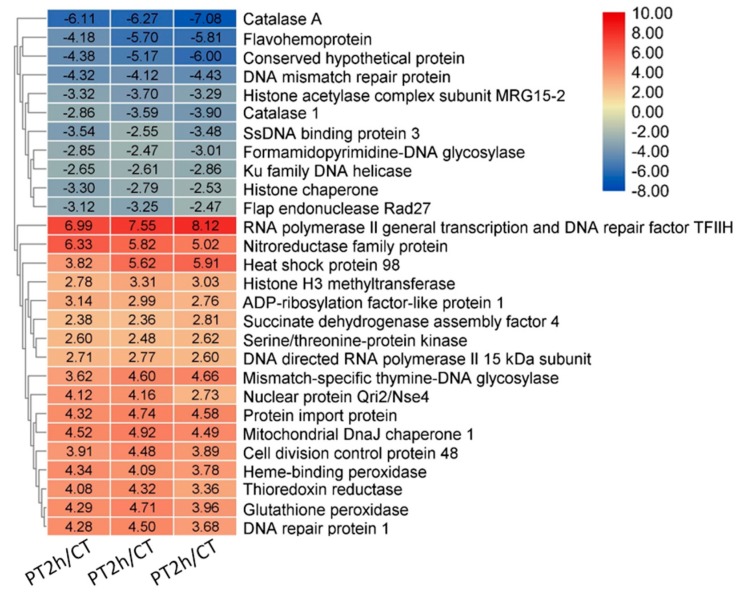
Heat map of differentially expressed genes in the response to stress term in PAE treatment and control groups.

The term of response to stress was remarkably enriched with an adjusted *p*-value = 1.41 × 10^−3^, and had 64 DEGs. Owing to many genes in this term, a rigid screening criterion of absolute values of log_2_ FoldChange > 2.5 was used to screen for pivotal genes associated to stress mechanism induced by PAE. Consequently, 28 DEGs were filtered, and result of data was shown in Figure 5. On one hand, there were some genes associated to DNA damage response, such as DNA mismatch repair protein, DNA repair protein 1, RNA polymerase II general transcription, and DNA repair factor TFII H, which is in accord with the term results described in above paragraph. On the other hand, many antioxidant genes were also enriched in this term. Remarkably, two genes of catalase were sharply downregulated, indicating scavenging activity of H_2_O_2_ negatively affected by PAE. However, note that two critical antioxidant genes, thioredoxin reductase, and glutathione peroxidase were remarkably upregulated. Thioredoxin reductase belongs to thioredoxin system, and can reduce H_2_O_2_ to water using thioredoxin [17]. Glutathione peroxidase could affect resistance of fungal cells to oxidative stress by accepting GSH, glutaredoxin, and thioredoxin as reducing substrate [18,19]. Besides, a gene encoding succinate dehydrogenase assembly factor also was upregulated, helping to produce reducing power (NADH). Therefore, it seems like the antioxidant activity in *A. flavus* is improved due to PAE treatment.

### 2.4. KEGG Enrichment Analysis of DEGs

**Figure 6:**
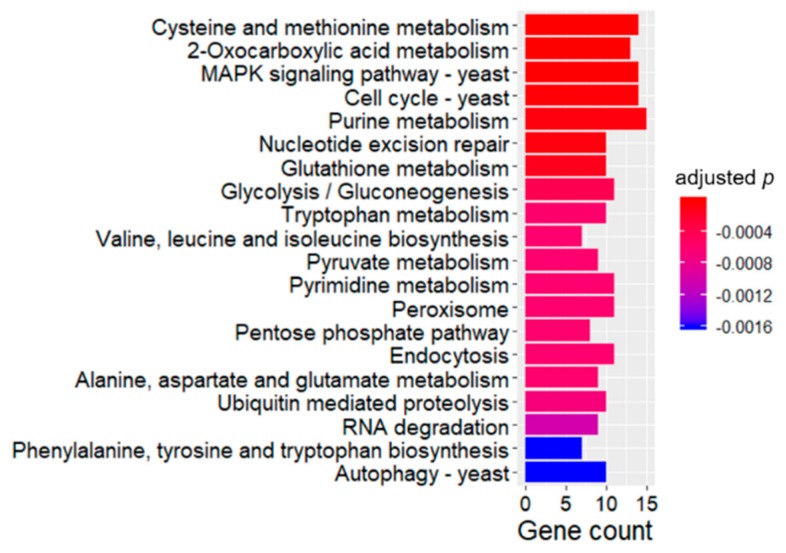
Enriched KEGG pathways of differentially expressed genes (DEGs). This figure shows top 20 pathways of KEGG enrichment analysis of common DEGs in PAE treatment and control groups.

KEGG is an efficient approach for network prediction of higher-level complexity of cellular processes and organism behavior [20]. Enrichment analysis of KEGG pathway was operated, and enriched terms were ranked by adjusted *p*-values, and closely related 20 terms in response to PAE treatment were shown on a bar plot in Figure 6. Consequently, four antioxidant pathways were screened. To begin with, a pathway of glutathione metabolism was significantly enriched. The pathway included two critical upregulated DEGs, namely glutathione peroxidase and glutathione oxidoreductase showing a direct function against oxidative damage [18,21]. Besides, two key glycometabolic pathways were enriched and might indirectly affect oxidative damage of *A. flavus* induced by PAE. Most DEGs belonging to these two pathways were downregulated, implying that reducing equivalent generation was restrained and antioxidant molecules were in an oxidation state with losing protective functions. In lieu of these given reasons, many antioxidant genes were upregulated in GO enrichment analysis as described in above text; *A. flavus* also suffered seriously oxidative damage and apoptosis as proved by our previous study [10]. In addition, the autophagy-yeast pathway was activated in response to energy stress induced by PAE. Finally, MAPK signaling pathway might play an important role in antioxidant response to PAE treatment.

### 2.5. A Protein-Protein Interaction Network Analysis of DEGs

**Figure 7:**
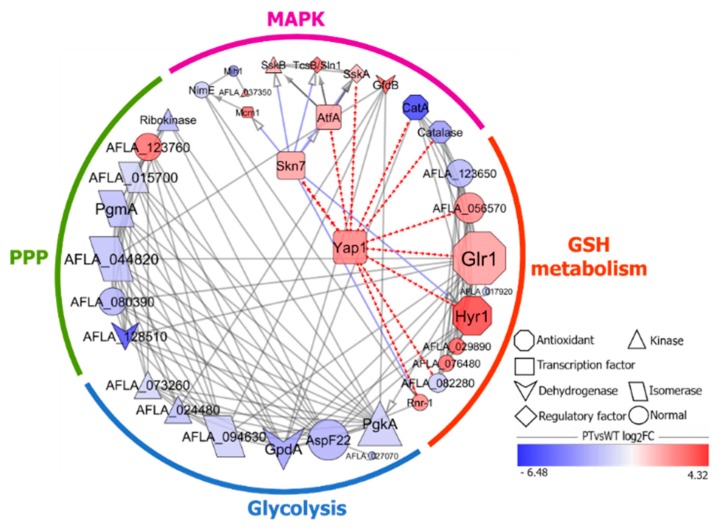
Protein-protein interaction network of differentially expressed genes. The icon size is proportional to the number of protein-protein interactions. Red fill color stands for positive regulation of proteins encoded by corresponding genes, and blue fill color for negative regulation of proteins.

Yap1 may take part in the regulation of response to oxidative stress of *A. flavus* induced by PAE. Yap1 serves as a pivotal transcription factor in response to oxidative stress in fungi, and has an ability to mediated expression of glutathione peroxidase [22] and glutathione oxidoreductase (Glr1) [23] enriched in the glutathione metabolism pathway. Two glycometabolic pathways, namely glycolysis and PPP, involve in production of NADPH: a metabolite required for oxidative stress resistance [15]. The MAPK pathway mediated cellular responses to various stimuli, including ROS [24]. In addition, Skn7 is also a transcriptional regulator that cooperates with Yap1 in response to redox stress signals [25]. Therefore, we hypothesized that Yap1 and Skn7 may play a pivotal role in stress resistance to PAE. Finally, the interactive relationship of 36 genes (Table 3) in GSH metabolism pathway (GshMP) containing both two transcriptional regulators and gene sets of four key pathways filtered through KEGG enrichment analysis above were analyzed by a protein-protein interaction network (Figure 7).

The interaction relationship of gene sets enriched in four key pathways was revealed in Figure 7 in details. In general, DEGs in four pathways were divided into two groups: one downregulated group included glycolysis and PPP pathway, and another upregulated group consisted of MAPK signaling pathway and GshMP.

First, two critical genes, *AFLA_073260* and *AFLA_024480*, encoding hexokinase and 6-phosphofructokinase, respectively, were remarkably downregulated in glycolysis pathway, and so conversion of glucose to fructose 1,6-bisphosphate was suppressed [26]. Moreover, triosephosphate isomerase encoded by *AFLA_094630* helped to generate glyceraldehyde-3-phosphate, which was conversed to 1,3-diphosphoglyceric acid by glyceraldehyde 3-phosphate dehydrogenase GpdA encoded by *AFLA_025100* coupled with NADH production. Besides, enolase AspF22 encoded by *AFLA_037480* catalyzed the generation of phosphoenolpyruvic acid, a critical substrate for ATP production. In short, given the genes above being all downregulated, therefore, the glycolysis process was roundly inhibited by PAE from glucose activation to reducing power (NADH) and energy (ATP) generation.

Secondly, the PPP was severely restrained by PAE. Initially, there was a rising trend of transcriptional regulation of glucose 6-phosphate, a primary substrate for PPP [27]. An accumulation of glucose-6-phosphate in glycolysis was induced by downregulation of glucose-6-phosphate isomerase encoded by *AFLA_044820* coupled with a conversion inhibition of glucose 6-phosphate to fructose 6-phosphate, and of phosphoglucomutase PgmA encoded by *AFLA_030710* inhibiting a conversion of glucose-6-phosphate to glucose-1-phosphate in gluconeogenesis. Thus, it seemed like the PPP acts as a key effector in response to the stress caused by PAE. Moreover, a following critical metabolic step of NADPH production in PPP was blocked by two sharply downregulated genes. In detail, 6-phosphogluconolactonase encoded by *AFLA_080390* catalyzed the formation of 6-phosphogluconic acid, which afterwards was dehydrogenized under a catalytic action of 6-phosphogluconate dehydrogenase encoded by *AFLA_128510*, coupled with producing numerous NADPH. In summary, it is likely that PPP is inhibited by PAE especially in the step of NADPH generation.

By contrast to the two downregulated glycometabolism pathways described above, transcriptional level of DEGs in GshMP was increased in an overall gene expression profile in response to PAE stress. It seemed like the negative expression of a glutathione synthetase encoded by *AFLA_123650* acts as a key player resulting in a direct decrease of GSH level. According to our data, glutathione peroxidase Hyr1 encoded by *AFLA_079910* was a dramatically upregulated antioxidant enzyme, of which expression was specifically induced in presence of Yap1 [22]. Besides, upregulated glutathione oxidoreductase Glr1 encoded by *AFLA_083370* could regenerate the reduced glutathione playing a critical role in cellular defense against oxidative damage [21], and it was also a target gene for Yap1 transcriptional regulation [23]. Moreover, glutathione S-transferase involves in resistance against oxidative stress [28], and two genes encoding glutathione S-transferase were sharply upregulated with a log_2_FoldChange value greater than 3. Finally, ribonucleotide reductase large subunit Rnr1 encoded by *AFLA_113250* was positively expressed in response to DNA damage [29]. Obviously, the antioxidant function of GshMP was enhanced. Perhaps, the decrease of reducing power content caused in glycolysis and PPP described above was the initial reason why an oxidative damage took place in *A. flavus* under PAE stress.

Autophagy serves as a survival strategy through cellular self-digestion in response to nutrient starvation, ubiquitously occurring in plant, animal, and fungal cells [30]. Severe inhibition of glucose metabolism was described above, and some DEGs were enriched in Autophagy pathway. Atg17 is a critical gene involving in autophagosome formation [31], and our data showed that this gene encoded by *AFLA_138020* was upregulated. Moreover, serine palmitoyltransferase 2 acts as apoptosis-inducing gene [32], and was also upregulated. Actually, autophagy can act as an approach to cell death through activating apoptosis pathway [33]. Together with our previous results that PAE can induce an apoptosis in *A. flavus* [10], autophagy may play a critical role in response to PAE stress.

As for MAPK pathway, functions of enriched DEGs were divided into three modules associated to various biological processes. The first functional gene group consisted of two downregulated H_2_O_2_-scavenging genes, namely, catalase and spore-specific catalase CatA, encoded by *AFLA_034380* and *AFLA_056170*, respectively. The second group of DEGs involved in osmotic stress response, and all genes were upregulated. Glycerol 3-phosphate dehydrogenase GfdB encoded by *AFLA_046760* was a NADP^+^-dependent glycerol dehydrogenase, and can respond to hyperosmotic shock in Aspergillus [34]. Besides, SskA encoded by *AFLA_062210* [35] was a response regulator to adapt to osmotic stress [36], and was regulated by AtfA [35], a bZIP transcription factor encoded by *AFLA_031340* regulating oxidative and osmotic stress responses in Aspergillus [37]. In HOG pathway, TcsB/Sln1 encoded by *AFLA_039130* was a sensor histidine kinase/response regulator, and regulated the downstream gene expression of SskB a MAP kinase kinase kinase [38], which responded to osmotic shock [39]. Interestingly, the third gene set participated in cell cycle control. There were two downregulated genes associated to cell cycle control, namely, a G2/M-specific cyclin NimE and a cell cycle control protein tyrosine phosphatase Mih1. In fact, these two genes above were regulated by Mcm1, a MADS box transcription factor encoded by *AFLA_090910* related with regulation of M/G1 phase of the cell cycle [40] and were regulated by the transcriptional regulator Skn7, also having a cell cycle-related role [41]. The gene expression profile of MAPK pathway showed a specific mechanism of *A. flavus* in which antioxidant activity became weak, cell cycle was stocked, and resistance to osmotic shock was improved.

### 2.6. Confirmation of DEGs by RT-qPCR

**Figure 8:**
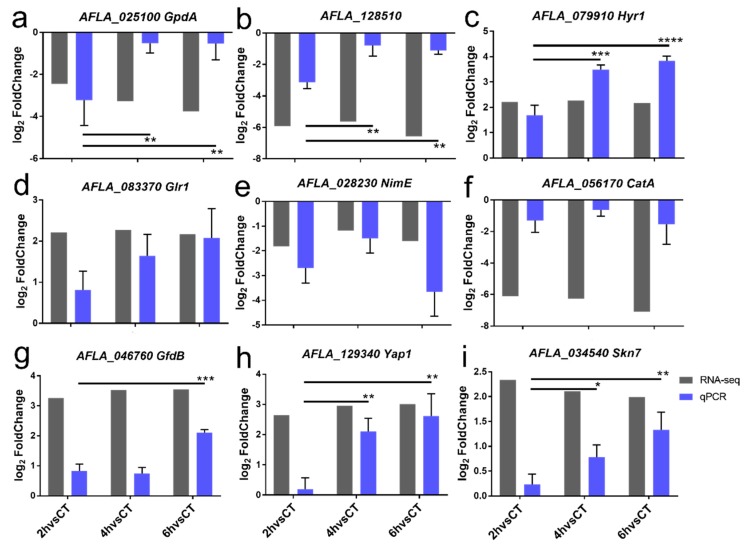
Validation of relative expression levels of critical differentially expressed genes in transcriptome data by RT-qPCR. (**a**) Glycolysis pathway, (**b**) 6-phosphogluconate dehydrogenase encoded by *AFLA_128510* of PPP pathway, (**c**,**d**) GSH metabolism pathway, (**e**–**g**) MAPK pathway, and (**h**,**i**) two transcription factors. Relative expression level of DEGs was showed using an index of log_2_FoldChange, which was calculated as a ratio of a gene expression level in PAE treatment and control group. Data are means ± standard deviation of three replicates. * *p* < 0.05; ** *p* < 0.01; *** *p* < 0.001; **** *p* < 0.0001.

To validate RNA-seq expression estimates, relative expression quantity of nine pivotal DEGs in four pathways described above were measured by RT-qPCR (Figure 8). According to present research, ROS accumulation is critical initiation of antifungal action of PAE [10]. In this research, RNA-seq data implied that inhibition of reducing power generation plays an ultimate role in bringing about oxidation damage of *A. flavus*. Therefore, first, we verified consistency of relative expression quantity of all dehydrogenase genes described in the protein-protein interaction network above, including *GpdA* of glycolysis pathway, 6-phosphogluconate dehydrogenase encoded by *AFLA_128510* of PPP pathway, and *GfdB* of MAPK pathway. Particularly, the downregulated expression of two dehydrogenase genes of *GpdA* and *AFLA_128510* led to an inhibition of reducing power generation. Second, two oxidative damage repair genes of *Hyr1* and *Glr1* of GshMP were upregulated, in accord with results of RT-qPCR assays. In addition, the upregulated expression of two transcription factors, also two antioxidant genes of *Yap1* and *skn7* were verified by RT-qPCR. These results highlighted the fact that activity of oxidative damage repair in *A. flavus* is enhanced by PAE stress. Besides, a result of RT-qPCR showed that expression of *NimE* associated to cell cycle control from MAPK pathway was restrained. Therefore, the results of RT-qPCR confirmed the reliability of RNA-seq data and our hypothetical antifungal mechanism.

## 3. Discussion

PAE seems to be a very attractive antifungal agent used for food storage. For a very long time now, PAE has been generally regarded as a safe flavoring ingredient for foods [5], and it has some hygienic functions, such as hypolipidemic action [42], protection against inflammation [43,44], and assisting in curing depression [45]. Besides, in our previous study, PAE showed an effective antifungal activity against some food-borne fungi, such as *A. falvus* [10], *A. niger* [9], and *Ceratocystis fimbriata* [46], with a striking inhibition of food decay, and against *Candida albicans*, which protects humans from candidiasis infection [44,47]. Moreover, the antifungal pathways of PAE must be partly explained. PAE, as a kind of EOs, may readily dissolve in biological membranes [48], and leads to a serious injury to plasma membrane through indirect interference in ergosterol biosynthesis [49], resulting in release of cytoplasmic contents into extracellular space. In fact, mitochondria is an important organelle consisting of phospholipid bilayers, and thus PAE can disturb its function, leading to abnormalities of the electron transport chain (ETC), resulting in producing numerous ROS [10]. Cyt c plays a critical role in cardiolipin oxidation, and then assists to release the proapoptotic Cyt c to cytoplasm [11]. Afterwards, metacaspases, orthologues of the caspase family in mammalian cells, are activated by proapoptotic Cyt c, and give rise to the cell death with apoptotic-like characteristics, such as phosphatidylserine exposure and DNA fragmentation in *A. falvus* [10]. In general, a metacaspase-dependent pathway seems to be a critical mechanism that PAE uses to induce *A. falvus* cell death.

The antifungal pathways associated to direct apoptotic processes have been revealed clearly; however, there is very little published research papers about the regulatory network of overall antifungal pathways based on a whole genome sequence data. To thoroughly address the antifungal mechanism, we used transcriptomics technologies to gain further insights into potential links between other biological processes and these antifungal pathways induced by PAE in *A. flavus*. In this assay, spores of *A. flavus* were treated by different concentration of PAE solutions. DEGs were judged by a comparison of RNA-seq data between experimental groups and control group. Afterwards, pathway enrichment analysis of GO and KEGG was used to analyze gene expression profiles of DEGs. Consequently, most of the topmost significantly enriched GO terms were found to be involved in stress response and DNA damage response, coinciding with our previous study results that PAE brought about ROS stress and DNA damage in *A. flavus* [10]. Intriguingly, KEGG enrichment analysis filtered four pathways in response to oxidant damage, namely, GSH metabolism, PPP, and glucolysis pathway, as well as MAPK signaling pathway. Therefore, we proposed that the antifungal mechanism of PAE is a complex regulatory network whereby metabolic inhibition of glucolysis and PPP plays an initial role in holding back generation of reducing power, which indirectly contributes to oxidant damage and subsequently apoptosis in *A. flavus*.

ROS contains superoxide anion (O_2_^−^), hydrogen peroxide (H_2_O_2_), and hydroxyl radical (HO·) et al. [50]. Numerous ROS may increase in response to external stimulus of Eos [51]. O_2_^−^ is generated by one-electron reduction of molecular oxygen (O_2_) through cytosolic NADPH oxidases (NOXs) or in mitochondrial ETC complexes [52]. However, the gene encoding NOXs in *A. flavus* showed a low-level expression with FPKM < 1, and so it was likely that NOXs do not participate in regulation of cell death induced by PAE. Consequently, ETC seemed to be the primary source of ROS production [51]. In matrix, $O_{2}^{-}$ is rapidly converted to H_2_O_2_ by superoxide dismutase (SOD) [52] and HO· is produced from H_2_O_2_ by Fenton reaction [53]. Given strong oxidizing potential, HO· may cause severe oxidative damage to cellular macromolecules, such as lipids, proteins and so on [54]. To combat oxidative stress, fungi have developed effective antioxidant systems consisting of GSH, glutathione and thioredoxin pathways [55]. Cysteine serves as a key residue in enzyme catalysis and protein fold et al., and is however easily oxidized by ROS, leading to unwanted modifications of proteins [56]. GSH abundant in matrix may reduce oxidized cysteine residues, and protects proteins from irreversible oxidation [21]. The oxidized GSH (GSSG) is recycled to reduction state by NADPH-dependent glutathione reductase (GR) [57]. Moreover, oxidized glutaredoxin is reduced by GSH [55], and formation of reduced thioredoxin is catalyzed by NADPH-dependent thioredoxin reductase [57]. Therefore, it is tempting to speculate that NADPH produced in PPP serves as the initial electron donor for antioxidant protection of fungi against EOs.

GSH metabolism may play a key role in response to PAE stress. In our previous assays, accumulation of ROS induced by PAE played a vital role in initiating an apoptosis process, coupled with large-scale DNA fragmentation that occurred in nuclear [10]. In line with this, using GO enrichment analysis, we screened many terms related to DNA damage response. Furthermore, we particularly revealed an insightful network of PAE antifungal pathways through KEGG enrichment analysis. First, due to the phenomenon of ROS accumulation, we energetically focused on the analysis of antioxidant pathways, especially GshMP. Unexpectedly, no inhibition effects emerging from GSH metabolism were responsible for PAE treatment, coupled with many upregulated antioxidant genes. In details, Glr1 glutathione oxidoreductase assists to regenerate GSH, and then Hyr1 glutathione peroxidase serves as a cofactor of the reduction reaction of oxidatively modified lipids and proteins, as well as glutathione S-transferase contributing to catalyzing a GSH-dependent reduction of organic hydroperoxides [58]. Besides, Rnr1, one of the major subunits of ribonucleotide reductase can protect cell against DNA damage by its repair function [59]. Except for these genes, Yap1, an overall regulator notably took part in the regulation of antioxidant processes with a large log_2_ FoldChange value. Apparently, GshMP serves as an antidrug resistance agent in *A. flavus* to PAE.

To further explore the causal relationship between *A. flavus* spore apoptosis and PAE stress, pathways generating reducing power were analyzed. Initially, we analyzed the regulation of genes in glycolysis. First of all, phosphorylation of glucose is catalyzed by hexokinases [60], and then 6-phosphofructokinase catalyzes a reversible generation of fructose 1,6-bisphosphate using fructose 6-phosphate [61]. The two genes above were downregulated in sequencing data. Afterwards, in the downstream pathway, the level of glyceraldehyde-3-phosphate decreased under the catalytic action of downregulated triosephosphate isomerase. During the following reaction process, an inhibition of NADH production occurred due to the lower-level reaction substrate glyceraldehyde-3-phosphate and negative regulation of catalyzing enzyme glyceraldehyde 3-phosphate dehydrogenase. Actually, NADPH serves as a key force needed for oxidative stress protection [62], of whose formation can be catalyzed by NADH kinase [16], and eventually, the antioxidant activity in *A. favus* decreases coupled with the low level of NADPH content. Moreover, the generation of another critical product ATP of glycolysis tended to be restrained due to downregulated expression of Enolase. Therefore, glycolysis process was nearly collapsed by PAE. On the other hand, PPP, a major source of reducing power NADPH [27] seemed to be also severely restrained by PAE. In the first place, 6-phosphogluconolactonase catalyzes the generation of 6-phosphogluconic acid, which is subsequently dehydrogenized under the catalytic action of 6-phosphogluconate dehydrogenase, coupled with numerous NADPH production. According to our data, the two genes described above were downregulated, indicating a committed generation step of NADPH was blocked. Therefore, the role played by NADPH in protection from oxidative stress [62] was suppressed. In conclusion, perhaps the inhibition of NADPH generation in glycometabolism plays a fateful role in triggering *A. flavus* spore apoptosis by PAE.

Besides, the MAPK pathway also played an important role in inducing *A. flavus* spore death by PAE. As we know, catalase is important in the detoxification of H_2_O_2_ [63], and so negative expression of two catalases in MAPK pathway revealed that *A. flavus* spores tend to suffer serious oxidant damage, especially under the condition of reducing power deprivation. On the other hand, there is an independent branch associated to cell cycle inhibition. Mih1 is a tyrosine phosphatase and can promote entry into mitosis by modification of dephosphorylation reactions [64]. NimE serves as a G2/M-specific cyclin [65]. The genes encoding the two important proteins above were all down regulated, indicating an inhibition action occurred in *A. flavus* spores. Except for the regulations of suppressing H_2_O_2_ elimination and cell proliferation, an osmotic response pathway was activated remarkably. First, GfdB is a NADP^+^-dependent glycerol dehydrogenase in Aspergillus in response to hyperosmotic shock [34]. Second, SskA acts as a response regulator related to osmotic stress [36], of which action is regulated by AtfA [35], a bZIP transcription factor regulating oxidative and osmotic stress responses in Aspergillus [37]. Besides, TcsB/Sln1 serves as a sensor histidine kinase/response regulator, and regulates the downstream gene expression of a MAP kinase kinase kinase SskB [38] in response to osmotic shock [39]. Surprisingly, ROS accumulation led to a noteworthy increase of osmotic response. Actually, this above relationship agreed with the research of Riddhiman and coworkers that yeast can adapt to a stressful environment by cross-protection, where oxidative stress protects against osmotic stress but not vice versa [66]. Therefore, MAPK pathway serves as an effector in inducing oxidant damage and cell cycle arrest.

## 4. Materials and Methods 

### 4.1. Chemical and Fungus Strain

The PAE (CAS Registry No. 18031-40-8) used in this study was purchased from Tokyo Chemical Industry Co. Ltd. PAE was dissolved in 1% (*v*/*v*) Tween-80 with ultrasonic wave, prepared as a mother liquor in a concentration of 5.0 µl/mL. *A. flavus* (NRRL 3357) was purchased from the China General Microbiological Culture Collection Center (CGMCC). The *A. flavus* was cultured for 4 days at 28 °C in potato dextrose agar medium (PDA, 20% potato, 2% dextrose, 1.5% agar) and stored in a refrigerator at 4 °C.

### 4.2. PAE Treatment and Sample Collection

The *A. flavus* spore suspension in 0.1% (*v*/*v*) Tween-80 was adjusted to 5 × 10^7^ spores/mL using a hemocytometer. Spore suspension and phosphate-buffered solution containing 2% glucose (PBS-2%G) were equivolumetrically mixed, incubated at 28 °C and 200 rpm for 4 h to obtain activated spores of *A. flavus*. After that, both 100 µL PAE mother liquor and 900 µL spore suspension were mixed to a final PAE concentration of 0.5 µL/mL. The spore suspension was cultured at 28 °C and 200 rpm. At treatment points of 2, 4, and 6 h, the fungal spores were harvested at 7000 g for 5 min and washed three times with PBS. All experiments were carried out in 3 independent biological replicates, and samples that were not treated with oil were used as a control treatment.

### 4.3. RNA Isolation, Library Construction, and Sequencing

RNA extraction was performed using Trizol (Invitrogen, Carlsbad, California, USA) according to the manufacturer’s instruction. RNA purity and concentration were assessed by Thermo Scientific NanoDrop 2000c, and RNA integrity was assessed with the Bioanalyzer 2100 RNA 6000 Nano Kit from Agilent Technologies. High-quality RNA was used in library construction. Sequencing libraries were generated using a NEBNext Ultra RNA library prep kit for Illumina (New England Biolabs, Ipswich, MA, USA) following the manufacturer’s instruction. In brief, mRNA was enriched from total RNA using NEBNext Oligo d(T)_25_ magnetic beads, and then the purified mRNA was randomly fragmented by addition of fragmentation buffer. Afterwards, first-strand cDNA was synthesized using a random hexamer primer and ProtoScript II Reverse Transcriptase on mRNA template. Subsequently, second-strand cDNA synthesis was performed using RNase H and DNA polymerase I, and then the double-stranded cDNA was purified using 1.8 X Agencourt AMPure XP Beads. The cDNA library was performed by End Repair/dA-tail, and immediately proceeded to adaptor ligation in preparation for hybridization, and then the ligation reaction was purified using AMPure XP Beads in order to obtain larger 200 nt size of fragments. After that, the cDNA library was enriched by PCR using NEBNext high-fidelity DNA polymerase, universal PCR primers and index (X) primer, and was purified using Agencourt AMPure XP Beads. Finally, the library quality was assessed using an Agilent Bioanalyzer 2100 system. Libraries were sequenced on an Illumina Hiseq X Ten platform.

### 4.4. Data Analysis

Raw reads were detected using a sequencing-by-synthesis technology, and they were stored in FASTQ format. Clean reads for downstream analysis were obtained by removing adapters and poor quality reads. Afterwards, the Q30 and GC contents of clean data were calculated. Clean reads were mapped to a reference genome of *A. flavus* NRRL3357 (assembly JCVI-afl1-v2.0) using TopHat2 software [67], and the number of reads mapped to each gene was counted. To measure the quantitative relation between mapped read counts and their real expression level, Cufflinks software was used to calculate for Fragments Per Kilobase of transcript per Million fragments mapped (FPKM) of each clean read based on mapped read counts and transcript length [68].

### 4.5. Differential Expression Analysis

Differentially expressed genes (DEGs) were screened by a comparison of PAE treatment groups with control groups and then analyzed with DESeq package using RStudio software (version 1.2.1335; RStudio, Inc.). To control false positive results in hypothesis test, the *p*-value was adjusted using the Benjamini–Hochberg approach to calculate the false discovery rate (*FDR*). DEGs were identified based on an adjusted *p* value < 0.01 and absolute value of log_2_ FoldChange ≥1. Moreover, to eliminate lower expressed DEGs, the numerous counts were screened with a stricter criterion of FPKM > 1 at each time-point after *A. flavus* spores were treated with PAE.

Expression patterns of DEGs were analyzed by Venn diagram, multidimensional scaling (MDS) plot, and heat map. First, differential expression numbers of DEGs at different PAE treating time points were showed by Venn diagram using VennDiagram package with RStudio, and then the common DEGs at each time-point were selected for subsequent analysis. Moreover, a MDS plot and a heat map of DEGs’ FPKM were drawn using limma package [69] and plots package [70] within RStudio, respectively.

Gene Ontology (GO) annotation and enrichment of DEGs were performed using clusterProfiler package using RStudio software [71]. The GO annotation file of *A. flavus* was retrieved with AnnotationHub package within RStudio. For functional enrichment analysis of DEGs, categories with an adjusted *p* value < 0.05 were counted as enriched term in GO term enrichment.

Kyoto Encyclopedia of Genes and Genomes (KEGG) enrichment and protein–protein interaction analysis were calculated by STRING v11 [72]. The gene list of DEGs was written in an analysis interface of multiple proteins, and then searched following the website instruction. KEGG pathways were enriched using the Analysis function, and only the pathways with *FDR* < 0.05 were counted as significantly enriched. On the other hand, protein–protein interaction data were outputted using the Exports function to a TSV file. Subsequently, the TSV file was imputed into Cytoscape (version 3.6.3), and then an aesthetic protein-protein interaction network was drawn.

### 4.6. Validation of DEGs by RT-qPCR

To confirm the reliability of RNA-seq data, relative expression quantity of 9 key DEGs in different pathways were measured by real-time qPCR (RT-qPCR). PCR primers were designed using Primer Premier 6 software (Premier Biosoft International, USA). The β-tubulin gene was used as an internal control for normalization of gene expression [73]. First-strand cDNAs were synthesized using a RevertAid First Strand cDNA Synthesis Kit (Thermo Scientific, Waltham, MA, USA). RT-qPCR was performed using a 2× Plus SYBR real-time PCR mixture Kit (BioTeke, Beijing, China) on an Applied Biosystems StepOnePlus™ Real-Time PCR system (Applied Biosystems, Foster, CA, USA). Each RT-qPCR mixture contained 1 μL template, 0.5 μL forward primer and 0.5 μL reverse primer, 10 μL 2× Plus SYBR real-time PCR mixture, 0.4 μL ROX1, and 7.6 μL DEPC-ddH_2_O. Thermal cycling parameters for RT-qPCR were as follows; 94 °C for 2 min, 40 cycles of 94 °C for 15 s, 60 °C for 30 s. Relative expression level was calculated using the 2^−ΔΔCt^ method [74].

### 4.7. Statistical Analysis

The statistical significance of data from different PAE treatment groups was calculated by one-way ANOVA with Duncan multiple range tests using SPSS 21.0 software (IBM-SPSS Inc., USA). * *p* < 0.05; ** *p* < 0.01; *** *p* < 0.001; **** *p* < 0.0001.

## 5. Conclusions

**Figure 9:**
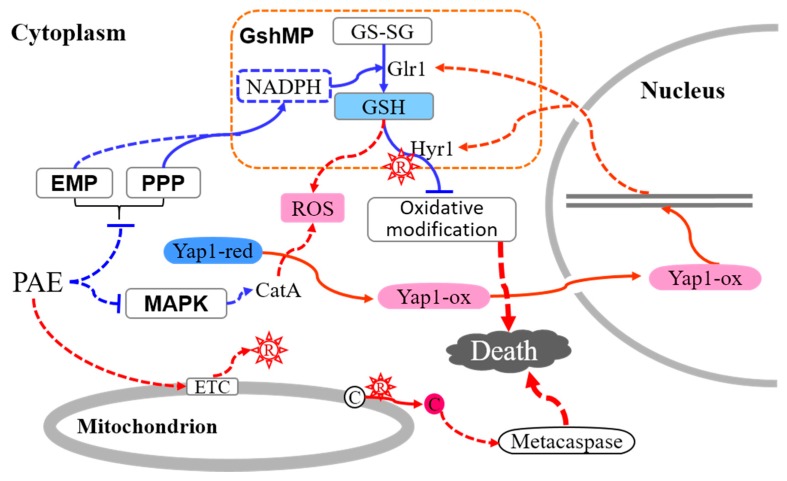
A schematic illustration of the potential mechanism that PAE induces cell death in *A. flavus*.

In conclusion, results of this study show a molecular regulation mechanism of PAE inducing cell death of *A. flavus* by primarily inhibiting energy metabolism (Figure 9). PAE exposure gives rise to an accumulation of ROS [10], which triggers an increase of antioxidant activity by activating GshMP and antioxidant transcription factor Yap1, to combat the oxidative damage. On the other hand, however, glucolysis and PPP are remarkably inhibited by PAE, coupled with a downregulated expression of key dehydrogenase genes, resulting in a less production of reducing power, which further inhibits the regeneration of GSH and sharply weaken the ability of preventing oxidative damage in *A. flavus* cells. Besides, MAPK pathway is also regulated by PAE via the negative expression of catalase *CatA*, with a result of a block of ROS scavenging, and a contribution to ROS accumulation, strengthening the oxidative damage. In the end, PAE induces superfluous ROS, initiates a metacaspase-dependent apoptosis pathway, and at last results in cell death of *A. flavus*. To thoroughly illuminate the antifungal mechanism of PAE mediated by inhibition of reducing power production, further researches need to pay more attention to specific regulation mechanism of glycometabolism pathways in *A. flavus* after PAE treatment.

## Figures and Tables

**Table 1:** Summary of sequencing data and mapped results in this study.

Samples	Duplicates	Raw Reads	Clean Reads	Clean Bases/Gb	Q30/%	Mapped/%
	1	14,463,254	14,010,225	3,528,805,818	88.10	86.06
CT	2	12,973,364	12,519,649	3,153,153,160	87.79	85.49
	3	12,891,043	12,502,271	3,148,706,828	88.17	86.23
	1	16,474,698	14,912,152	3,754,934,036	88.70	90.18
PT2h	2	15,322,010	13,912,871	3,503,972,558	88.41	89.78
	3	16,769,520	15,215,838	3,832,001,194	89.98	90.31
	1	18,805,595	17,107,102	4,308,390,052	89.92	90.27
PT4h	2	17,976,647	16,481,808	4,150,736,108	90.18	90.55
	3	18,935,548	17,335,243	4,365,533,832	89.94	89.73
	1	16,979,324	13,183,354	3,319,958,436	85.43	84.33
PT6h	2	17,624,059	15,768,269	3,971,226,468	90.00	89.71
	3	21,925,695	19,631,355	4,942,988,542	89.96	89.82

CT: control treatment group; PT2h: PAE treatment group for 2 h; PT4h: PAE treatment group for 4 h; PT6h: PAE treatment group for 6 h.

**Table 2:** Variation trend of gene expression quantities of differentially expressed genes (DEGs) in different comparisons.

Comparisons	Up	Down	Total
PT2h/CT	1848	950	2798
PT4h/CT	1928	1024	2952
PT6h/CT	1872	1082	2954

**Table 3:** Representative DEGs in different comparisons between each PAE treatment and control group.

Pathway	PT vs. CT log_2_ Fold Change			Name	Product
	2 h	4 h	6 h		
Glycolysis					
*AFLA_073260*	−1.09	−1.88	−1.08		hexokinase
*AFLA_024480*	−1.49	−1.76	−2.75		6-phosphofructokinase alpha subunit
*AFLA_025100*	−2.45	−3.28	−3.76	*GpdA*	glyceraldehyde 3-phosphate dehydrogenase
*AFLA_027070*	−2.35	−2.43	−2.06		acetate-CoA ligase
*AFLA_037480*	−2.52	−3.22	−1.96	*AspF22*	enolase
*AFLA_069370*	−1.26	−1.70	−1.77	*PgkA*	phosphoglycerate kinase
*AFLA_094630*	−1.15	−1.68	−1.68		triosephosphate isomerase
PPP					
*AFLA_080390*	−2.12	−1.96	−3.27		6-phosphogluconolactonase
*AFLA_030710*	−1.65	−2.02	−2.65	*PgmA*	phosphoglucomutase
*AFLA_015700*	−1.31	−1.01	−1.78		ribulose-phosphate 3-epimerase
*AFLA_044820*	−1.83	−2.13	−2.10		glucose-6-phosphate isomerase
*AFLA_123760*	2.84	4.39	2.76		deoxyribose-phosphate aldolase
*AFLA_125700*	−2.70	−2.24	−2.02		ribokinase
*AFLA_128510*	−5.91	−5.62	−6.57		6-phosphogluconate dehydrogenase
GSH metabolism					
*AFLA_076480*	3.45	3.27	2.73		glutathione S-transferase
*AFLA_079910*	4.29	4.71	3.96	*Hyr1*	glutathione peroxidase
*AFLA_082280*	−1.22	−1.80	−1.69		glutathione S-transferase
*AFLA_083370*	2.21	2.27	2.17	*Glr1*	glutathione oxidoreductase
*AFLA_029890*	3.06	3.62	3.81		glutathione S-transferase
*AFLA_017920*	−1.75	−1.55	−1.82		spermidine synthase
*AFLA_113250*	2.43	2.98	2.44	*Rnr-1*	ribonucleotide reductase large subunit
*AFLA_056570*	2.75	2.61	2.44		glutathione synthetase
*AFLA_123650*	−2.95	−1.12	−2.95		glutathione synthetase
MAPK					
*AFLA_028230*	−1.82	−1.18	−1.60	*NimE*	G2/M-specific cyclin
*AFLA_031340*	1.98	2.27	2.01	*AtfA*	bZIP transcription factor
*AFLA_034380*	−2.86	−3.59	−3.90		catalase
*AFLA_037000*	−3.04	−3.9	−3.88	*Mih1*	cell cycle control protein tyrosine phosphatase
*AFLA_037350*	2.05	2.07	2.31		casein kinase I
*AFLA_039130*	2.58	3.49	3.60	*TcsB/Sln1*	sensor histidine kinase/response regulator
*AFLA_046760*	3.27	3.54	3.55	*GfdB*	glycerol 3-phosphate dehydrogenase
*AFLA_056170*	−6.11	−6.27	−7.08	*CatA*	spore-specific catalase
*AFLA_062210*	1.75	1.65	1.20		response regulator
*AFLA_068590*	1.71	1.97	1.57	*SskB*	MAP kinase kinase kinase
*AFLA_090910*	2.81	3.61	3.57	*Mcm1*	MADS box transcription factor
*AFLA_129340*	2.65	2.95	3.01	*Yap1*	AP-1-like transcription factor
*AFLA_034540*	2.34	2.11	1.99	*Skn7*	stress response transcription factor

## Abbreviations

Cyt c	cytochrome c
DEGs	differentially expressed genes
EOs	essential oils
ETC	electron transport chain
FPKM	fragments per kilobase of transcript per million fragments mapped
GO	Gene Ontology
GSH	glutathione
GshMP	GSH metabolism pathway
KEGG	Kyoto Encyclopedia of Genes and Genomes
MAPK	mitogen activated protein kinase
MDS	mitogen activated protein kinase
MDS	multidimensional scaling plot
NADPH	reduced nicotinamide adenine dinucleotide phosphate
NOXs	NADPH oxidases
PAE	perillaldehyde
PPP	pentose phosphate pathway
RT-qPCR	real-time qPCR
ROS	reactive oxygen species
Trx	thioredoxin
